# Influence of spent mushroom substrate and molasses amendment on nitrogen loss and humification in sewage sludge composting

**DOI:** 10.1016/j.heliyon.2020.e04988

**Published:** 2020-09-22

**Authors:** Liqiang Meng, Weiguang Li, Xiancheng Zhang, Yi Zhao, Li Chen, Shumei Zhang

**Affiliations:** a Institute of Microbiology, Heilongjiang Academy of Sciences, 150010, Harbin, China; b Institute of Advanced Technology, Heilongjiang Academy of Sciences, 150020, Harbin, China; c School of Environment, Harbin Institute of Technology, 150090, Harbin, China; d State Key Laboratory of Urban Water Resource and Enviroment, Harbin Institute of Technology, 150090, Harbin, China

**Keywords:** Environmental analysis, Environmental engineering, Environmental management, Environmental pollution, Biotechnology, Compost, Spent mushroom substrate, Molasses, Sewage sludge, Nitrogen loss, Maturity

## Abstract

The present study included lab-scale sewage sludge (SS) composting amended by molasses and spent mushroom substrate (SMS) in 5 L composting reactor system. The influence of molasses and SMS amendment on nitrogen loss and humification of SS composting was evaluated. The results showed that SMS amendment, especially combination with molasses raised composting temperature, increased CO_2_ volatilization, promoted organic matter degradation, improve germination index and humification process. The addition of SMS and molasses contain carbohydrates used as carbon source and energy substance by microorganisms could increase microbial activity and ammonia assimilation. In the SMS + molasses treatments, NH_3_ volatilization was reduced by 33.1%–37.3% and N_2_O volatilization was only 17.8%–25.4% of that in the control treatment, furthermore, the nitrogen loss rate was reduced by 27.2%–32.2%. Consequently, the addition of SMS and molasses improved the compost maturity and reduced nitrogen loss in the SS composting process.

## 1 Introduction

With the rapid development of municipal wastewater treatment facility, approximately 44 million tons of sewage sludge (SS) were produced every year in China, and the quantity increased by 13% every year (Cai et al., 2016; Guo et al., 2019). If the SS is not managed properly, it will cause serious secondary environmental pollution, such as heavy metal, pathogenic microorganism, organic micro-pollutant, and so on (Wang et al., 2017). Consequently, it's very critical to research economical and practical techniques for treatment and disposal of SS.

Aerobic composting is an effective and cost-efficient biotreatment technology to transform SS into stabilized fertilizer. However, SS is not suitable for composting alone owing to its high moisture content and small particle, so it must be mixed with dry material as bulking agents to improve the free air space of composting materials. Lots of studies have evaluated the effects of bulking agents, such as sawdust, barley straw, eggplant waste and spent mushroom substrate (Kulikowska and Sindrewicz, 2018; Meng et al., 2018a; Toledo et al., 2019; Wang et al., 2018). In addition, the low C/N ratio of SS will lead to the serious nitrogen loss caused by N_2_O and NH_3_ volatilization in the composting process. The nitrogen can be recycled for the plant growth, so it is very important to keep the nitrogen in the composting endproduct. However, about 40–80% of nitrogen was lost through NH_3_ volatilization in the SS composting, which has been reported in the previous reports (Li and Li, 2015; Nakhshiniev et al., 2014). Moreover, “*Kyoto Protocol*” reported that N_2_O is a key greenhouse gas, that play a great role in the global warming, and its single-molecule warming potential is 296 times that of CO_2_ (IPCC, 2013). Therefore, it is necessary to reduce the NH_3_ and N_2_O volatilization in composting process to control nitrogen loss and alleviate the environmental impacts.

The present study involves the influence of spent mushroom substrate (SMS) and molasses amendment on the SS composting. The SMS is a by-product of edible fungus production, and the production of SMS about 2.5 million tons yearly in Heilongjiang Province alone (Meng et al., 2019). If the SMS is not properly disposed, it will waste resources and pollute the surrounding environment (Gao et al., 2015; Hu et al., 2019). The SMS could serve as bulking agent in SS composting, in virtue of its low bulk density and moisture content. Moreover, SMS contains enzymes that promote biochemical reactions in the process of organic matter (OM) degradation, such as laccase, protease, hemicellulase and cellulase (Bian et al., 2017).

It's found that the addition of carbon source material in SS composting could control the NH_3_, N_2_O volatilization and reduce the nitrogen loss (Li et al., 2017; Meng et al., 2016b). It's proved that readily degradable carbon sources (e.g. glucose and sucrose) amendment could improve the ammonia assimilation of microorganism and accelerate the transition of NH_4_^+^-N to bio-nitrogen, so reduced NH_3_ and N_2_O volatilization (Meng et al., 2016a, 2018a). However, due to the consideration of production costs in the actual production process, it is necessary to look for the a low-cost material as substitute for glucose and sucrose. Beet molasses is by-product in sugar produced industry, whice is a dense, darkly coloured substance with composition of sucrose vitamins and minerals (Torkashvand, 2009). Molasses is a potential substitute for glucose and sucrose, but its effect on nitrogen conversion and composting process is unknown (Gabra et al., 2019).

Few studies of the use of SMS and molasses as extra carbon sources amendment in SS composting have been performed (Liang et al., 2006; Meng et al., 2017). So, the main purpose of present research was to evaluate the influence of molasses and SMS amended on physical-chemical properties, ammonia volatilization, N_2_O volatilization, nitrogen loss, humification and germination index (GI) in SS composting.

## 2 Materials and methods

### 2.1 Properties of compost substrate

SS was collected from Wenchang sewage disposal factory in Harbin, China. Pumice used as inert bulking agent is a volcanic rock contain almost no organics and moisture. It's responsible for increasing the ventilation property of the composted material in control treatment. SMS of *Auricularia auricula* cultivation was collected from Fermentation Engineering Technology Center of Heilongjiang Province. SMS was comminuted by a grinder to become graininess and its moisture content was adjusted to 10–15% by proper airing. Beet Molasses purchased from Institute of Beet, Heilongjiang university, and then kept in the refrigerator until used. The characteristics of raw materials were shown in Table 1.

**Table 1:** The characteristics of the composting materials.

	SS	Pumice	SMS	Molasses
MC (%)	79.84	0.84 ± 0.08	12.44 ± 1.56	23.50 ± 1.01
OM (%)	62.44 ± 1.25	0.48 ± 0.11	80.54 ± 2.19	88.25 ± 0.55
pH	7.05 ± 0.14	7.15 ± 0.25	6.45 ± 0.47	4.87 ± 0.26
C/N	7.89 ± 0.32	—	37.22 ± 1.15	98.25 ± 2.55

SS = sewage sludge, SMS = spent mushroom substrate, MC = moisture content, OM = organic matter. The results are the mean ± standard deviation (n = 3).

### 2.2 Experimental system and protocol

Initially 2000 g of fresh dewatered SS was mixed with 600 g of SMS (30% wet basis), different dosages of molasses were added into experimental treatments P2, P3, P4 and P5 as shown in Table 2. The control treatment was just the mixture of 1000 g of pumice and 2000 g of fresh SS without SMS or molasses amended denoted as P1. The moisture content (MC) of mixtures was also adjusted to 60~63% by adding distilled water before starting the composting reaction. Five piles of compost materials were put into respective reactors with 5 L volume, whose detailed informations about reactors have been reported in authors' previous literature (Meng et al., 2017). In order to simulate the real composting process and reduce heat loss of laboratory-scale compost experiments, the reactor system was put into the same waterbox (Meng et al., 2016a). Composting experiment were forced to supply oxygen by an air pump, the air transport speed was set at 0.4 L/min. Air entered reactors through porous plates in the bottom after going through 10% NaOH solution to remove CO_2_.

**Table 2:** The dosage of amendments in five treatments (% on wet weight basis).

Treatments	Pumice	SMS	M (Molasses)
P1: Control	50	0	0
P2: SMS	0	30	0
P3: SMS+2%M	0	30	2
P4: SMS+4%M	0	30	4
P5: SMS+6%M	0	30	6

### 2.3 Sampling and analytical methods

The compost experiments were conducted for 20 days, and the samples were collected from upper, middle and lower part of every reactor on Day 0, 2, 4, 6, 8, 10, 14 and 20, respectively. After mixed well, the sample (approximately 20 g) was divided into two parts on average: one part was used to determine the moisture content by an oven, and then the dried samples were used to determine OM contents and total N; the remaining samples was stored at 4 °C for determing the pH, germination index (GI), fulvic acids (FA) and humic acids (HA).

The temperature was measured by thermometer inside the composting reactors, and recorded every hour. NH_3_ in the end gas was absorbed by 500 mL of 2% boric acid, then detected by titration (Li et al., 2013). 200mL end gas was collected by an aluminium sampling bag everyday according to the method of Xi et al. (2012). The contents of N_2_O and CO_2_ were detected by a gas chromatography followed the procedure of Luo et al. (2014). The content of OM in dried samples was detected by burning samples in muffle furnace at 550 °C for 5 h. The total N was deteced by an element analyser (Vario EL, Germany). The pH of sample was measured by a pH meter based on the water extract of samples that formed through sample dissolved in distilled water at weight rato 1/10. The extraction and determination of HA and FA used the methods of Wang et al. (2013). GI of samples was detected with cucumber seeds used the methods of Meng et al. (2017). The statistical analyses were completed by SPSS 16.0.

## 3 Results and discussion

### 3.1 Temperature and pH

**Figure 1:**
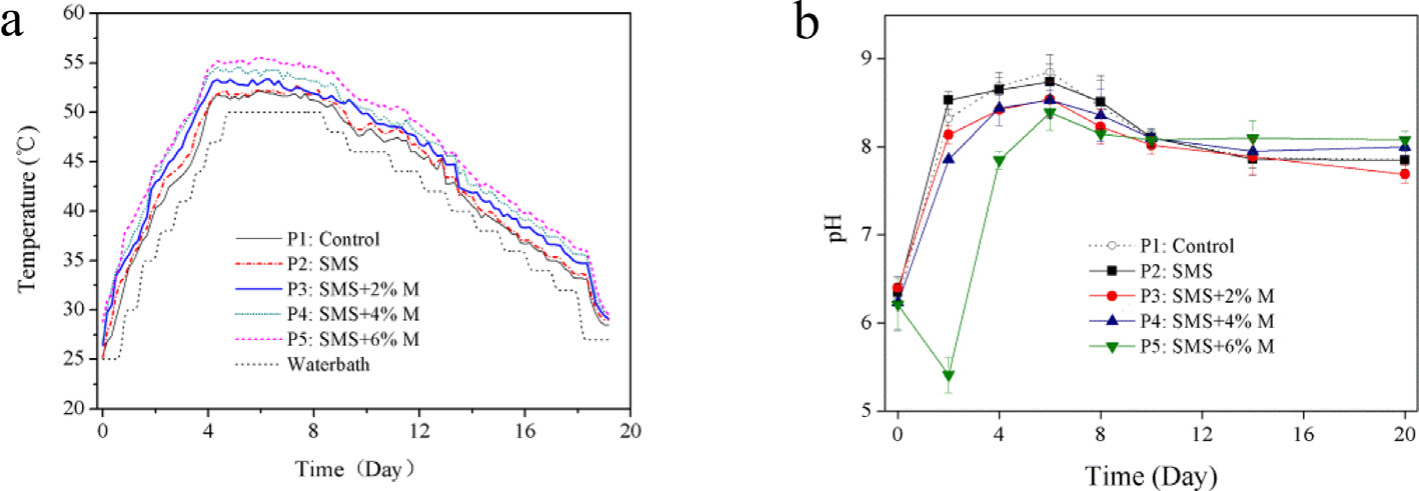
Evolution of temperature (a) and pH (b) in five treatments.

Temperature plays a important role in the composting progress. The temperature variation of each treatment in present study showed three typical composting phases, namely mesophilic phase, thermophilic phase and cooling phase, as shown in Figure 1a. In the first 4 days of composting, all treatments rapidly heated up and entered the thermophilic phase (over 50 °C). The addition of SMS increased the composting temperature, especially the combination of SMS and molasses in P3~P5. The temperature peaks of P1~P5 were 52.1 °C, 52.7 °C, 53.2 °C, 54.6 °C and 55.5 °C, and the maintenance times of thermophilic phase were 4.8, 5.2, 6.0, 6.7, and 7.7 days, respectively. The thermophilic phases of present study were shorter than those of previously literature (Maeda et al., 2018; Santos et al., 2016). This phenomenon was mainly caused by the difference of composting period and reactors sizes whose reactor could maintain longer thermophilic phase in the larger than smaller one (Pagans et al., 2006). The temperature was directly affected by the activity of microorganism and the amount of degradable OM in composting. SMS amendment, especially the combined SMS and molasses amendment, increases the content of easily degradable OM in the material, and also increases the activity of composting microorganism. More biological heat were produced in P2~P5 than that of P1 treatment due to pumice in P1 without any OM, therefore the temperatures of P2~P5 were higher than that of P1. In addition, the higher content of molasses was added, the higher temperature of composting was obtained. For example, the P5 treatment added 6% of molasses had the highest temperature peak, and the longest time maintained in the thermophilic phase. Obviously, the addition of molasses in P3~P5 increased the activity of microorganism and the degradation rate of OM in composting, so raised composting temperature. During the cooling phase, the temperature of each treatment gradually decreased to room temperature with the exhaustion of OM, and there was no significant difference between the treatments.

The pH value of the composting directly affects the activity of microorganisms and the form of nitrogen in composting, what's more, it is also one key factor affecting nitrogen loss (Awasthi et al., 2016). Excessively high pH value (over 8.5) in the composting material will cause NH_4_^+^-N to form NH_3_ and volatilize to the air, while excessively low pH (under 5.0) will reduce the bacterial activity and limit the OM degradation (Wong et al., 2017). The variations of pH of five treatments are shown in Figure 1b. The variation trend of pH value of each treatment is similar, all of them increased rapidly in mesophilic phase, then got to the maximum during thermophilic phase. Except for P5 treatment, showed a short-term acidity on Day 2 (pH: 5.41), that as a result of acidic intermediate products produced from the degradation of excess easily degradable carbon source by microorganisms, and then the acidity gradually disappeared with increasing temperature. The pH value of each treatment reached its maximum on the Day 6 in thermophilic phase, and the highest pH was observed in the control treatment P1 (8.85), followed by P2 (8.74), P3 (8.54), P4 (8.53), and P5 (8.39). Obviously, the addition of molasses reduced the pH value, and the reduction only exists in the thermophilic phase of the composting. With the volatilization of NH_3_ and the decrease of the temperature, the difference in pH among the treatments gradually disappeared. The pH value of each treatment gradually decrease in the cooling phase and remained neutral pH (7.9) until the end of composting implied that composting got to a stable state (Awasthi et al., 2016).

### 3.2 OM degradation and CO_2_ volatilization

**Figure 2:**
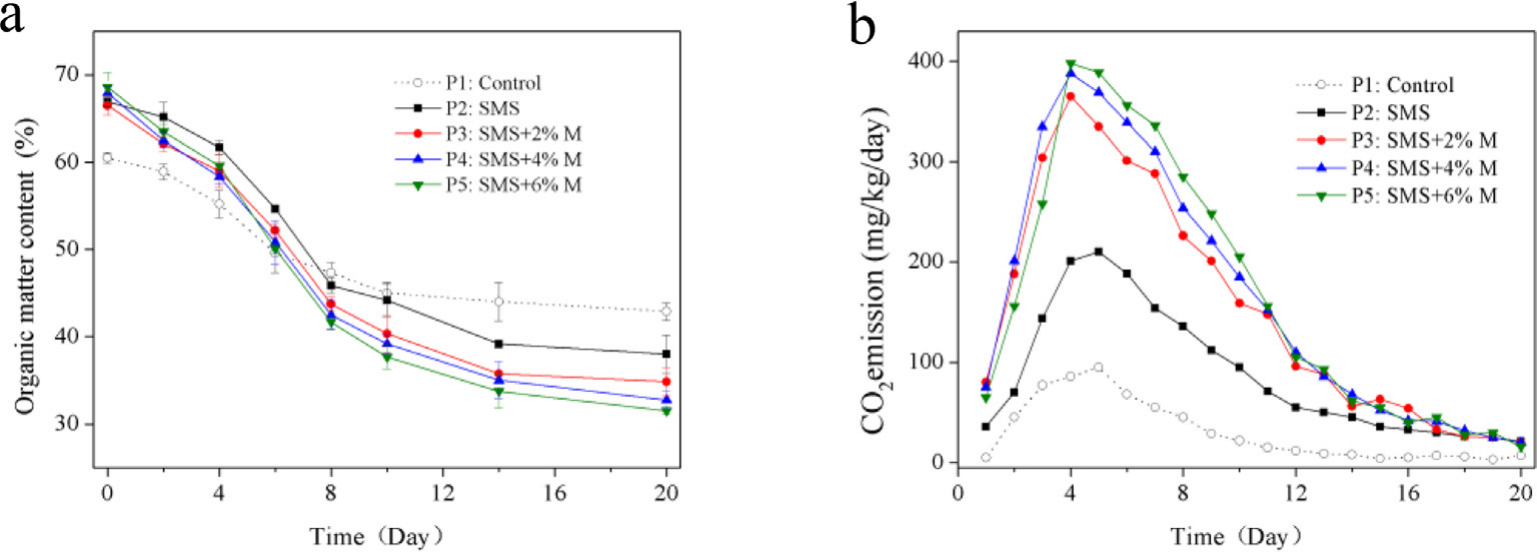
Evolution of OM degradation (a) and CO_2_ volatilization (b) in five treatments.

Figure 2a shows that initial OM contents in all SMS treatments were higher than that of the control treatment P1 whose bulking agent, pumice as an inert matter, does not contain any OM. The OM of SMS treatments rapidly decreased in mesophilic phase and thermophilic phase, especially in SMS + M treatments. In first 10 days, the OM of P1~P5 dropped to 45.1%, 42.9%, 38.4%, 37.3% and 35.2%, respectively, whose loss of OM were 15.5%, 27.3%, 31.4%, 34.5%, and 37.1%. Obviously, SMS and molasses amendment improved the degradation of OM. According to the literature SMS contains numerous enzymes that could promote OM degradation, therefore the OM loss in P2–P5 was larger than that in P1 (Zhang and Sun, 2014). The main component of the molasses is sucrose, could be used by microorganisms, so molasses could stimulate microbial activity (Liang et al., 2006; Meng et al., 2016b). Accordingly, the OM degradation rates in P3~P5 were faster than other treatments (without molasses amended). In addition, more biological heat was generated in SMS + M treatments whose composting temperatures were higher. And high temperature stimulated cellulose and hemicellulose degradation, because the actinomycetes would be dominated and active at high temperature (Kulikowska and Klimiuk, 2011). By the end of composting, the OM contents in P1~P5 treatments were 42.9%, 35.6%, 31.8%, 29.3%, and 27.8%, the lowest appeared in SMS+6%M treatment and the highest appeared in the control treatment, so the addition of SMS and molasses reduced the OM contents of the final product.

Biodegradation of OM resulted in the production of CO_2_ that have close relationship of respiratory chain in microbial cells (Cho et al., 2016). So, the volatilization of CO_2_ reflected degradation of OM and activity of microbial metabolic. The evolutions of CO_2_ volatilization are showed in Figure 2b. In the early phase of composting, due to the rapid degradation of OM, the CO_2_ volatilization rate of each treatment increased rapidly and reached maxima during thermophilic phase. Concretely, P1 and P2 treatment reached maximum of CO_2_ volatilization on the Day 5 of composting, which were 95.3 mg/kg/day and 210.7 mg/kg/day, respectively. Nevertheless, the maxima of CO_2_ volatilization in P3~P5 molasses amended appeared on the Day 4 of composting, which was one day earlier than that of P1 and P2 treatments, were 365.1 mg/kg/day, 388.0 mg/kg/day, and 398.8 mg/kg/day, respectively, which were significantly higher than the other two treatments. The addition of SMS could improve the porosity of composting materials and the transfer rate of oxygen, thereby provide a maximum microenvironment for microorganisms growth, increase the metabolic capacity of microorganisms, ultimately promote OM degradation and CO_2_ volatilization. Molasses contains abundant carbohydrate which could provide microorganisms with energy substances to accelerate their growth and stimulate their activity, and also promote OM degradation of and CO_2_ volatilization. Therefore, the amounts of CO_2_ volatilization in P3~P5 treatment was significantly higher than that of the control treatment P1. Similar observations were found by Meng et al., 2016a and Cai et al. (2016) who reported that the carbon source material amendment could promote OM degradation and CO_2_ volatilization.

### 3.3 NH_3_ and N_2_O volatilization

**Figure 3:**
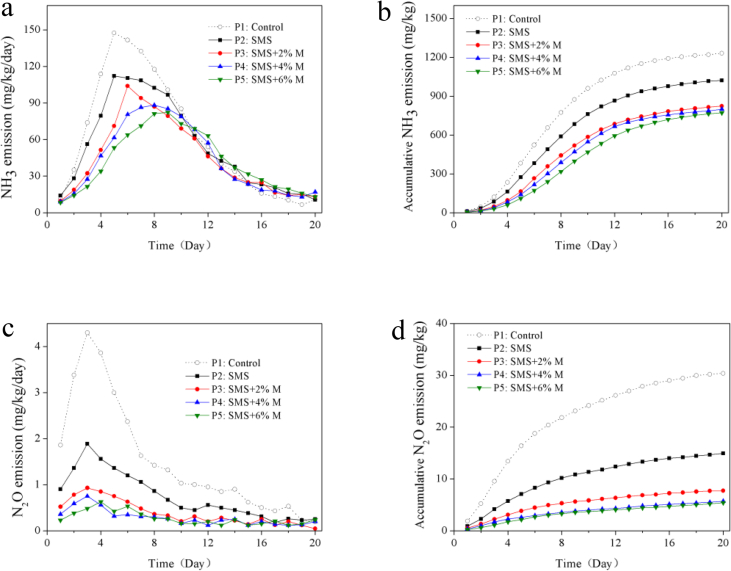
Evolution of NH_3_ volatilization (a), cumulative NH_3_ volatilization (b), N_2_O volatilization (c) and cumulative N_2_O volatilization (d) in five treatments.

Approximately 40–80% of the nitrogen loss is caused by the volatilization of NH_3_ in the SS composting process, which contains many nitrogen conversion reactions (Li and Li, 2015; Sasaki et al., 2005). Organic nitrogen compound could be degraded to inorganic nitrogen (mainly NH_4_^+^-N) by microbial ammonification in the mesophilic and thermophilic phase of composting and then escape from the composting system in the form of NH_3_. The authors' previous research proved that easily degradable carbon sources can enhance the ammonia assimilation of microorganisms and transform of NH_4_^+^-N to bio-nitrogen, thereby reduce the transform of NH_4_^+^-N to NH_3_ and the amount of NH_3_ volatilization (Meng et al., 2016a). The evolutions of NH_3_ volatilization of the five treatments are shown in Figure 3a. The NH_3_ volatilization of all treatment increased rapidly in the mesophilic phase, then got to respective maximum during thermophilic phase. P1 and P2 reached the maximum on the Day 5 of composting, which were 147.4 and 110.4 mg/kg/day, respectively. However, the maxima time of P3~P5 amended with SMS and molasses were slightly later than that of treatment control P1, appeared on Day 6, Day 8, Day 9, and were 102.3 mg/kg/day, 85.8 mg/kg, 79.8 mg/kg/day, respectively.

The addition of SMS and molasses inhibited the volatilization of NH_3_, furthermore the addition of molasses delayed the NH_3_ volatilization time, and the length of delay time was positively correlated with the amount of molasses amended. Similar phenomena have been reported in previous literature that easily degradable carbon source (glucose, sucrose) amendment inhibited and delayed the volatilization of NH_3_ (Meng et al., 2016b; Li et al., 2013). The cumulative volatilizations of NH_3_ of each treatment were shown in Figure 3b. The biggest NH_3_ volatilization appeared in the control treatment P1 was 1230.9 mg/kg. The addition of SMS significantly reduced the NH_3_ volatilization, which reduced 17.1% in the P2 than that of control treatment. Especially, the volatilization amounts of NH_3_ in P3~P5 treatment with the combined amendment of SMS and molasses, were reduced by 33.1%–37.3%, nevertheless, the difference among P3, P4 and P5 was not significant.

Although the nitrogen loss caused by N_2_O volatilization is much less than that caused by NH_3_ volatilization during the SS composting process, the greenhouse effect caused by N_2_O could not be ignored (Jiang et al., 2018). N_2_O is generally generated by nitrification and denitrification in the SS composting, and the main path of N_2_O production is denitrification which generally occurs in the area of insufficient oxygen or local anaerobic area of the reactor (Wu et al., 2018). Meanwhile, nitrification can generate N_2_O when the degradable OM is short and the oxygen is ample in the composting (Pan et al., 2018).

Figure 3c shows the evolution of N_2_O volatilization, the N_2_O volatilization rate of each treatment increased rapidly in the mesophilic phase, especially the control treatment reached a peak of 4.32 mg/kg/day on Day 3, which was much higher than those of other treatments, followed by P2 (1.89 mg/kg/day), P3 (0.93 mg/kg/day) and P4 (0.75 mg/kg/day). However, the minimum volatilization of N_2_O in P5 treatment was 0.63 mg/kg/day on Day 4 which later one day than the other treatments. So, the addition of SMS significantly reduced the volatilization of N_2_O. This might be due to the loose structure of SMS that increased the porosity of the composting material, increased the mass transfer rate of oxygen, and reduced the anaerobic area of the composting, thereby reduced the denitrification and the N_2_O volatilization generated by it. Similar observations were reported by Zhang and Sun (2014), who found that addition of 35% SMS in green waste composting could provide a suitable free air space for composting microorganisms. The volatilization reduction effects on N_2_O in the treatments P3~P5 amended with SMS and molasses were better than that in the treatment P2. The N_2_O volatilization of P3~P5 treatments only reached a small peak in mesophilic phase and thermophilic phase, then keep a low level in cooling phase.

The cumulative N_2_O volatilization of each treatment is shown in Figure 3d. The maximum N_2_O volatilization appeared in the control treatment was 30.37 mg/kg, followed by P2 at 14.93 mg/kg, P3 at 7.72 mg/kg, and P4 at 5.70 mg/kg, P5 is 5.38 mg/kg. The cumulative volatilization of N_2_O in P2 was 49.2% of that in control treatment. In addition, the cumulative N_2_O volatilization in P3~P5 treatment amended with SMS and molasses was only 17.8%–25.4% of that in the control treatment, nevertheless, the difference among the molasses amended treatments was not significant. Thus it follows that combined SMS and molasses amendment could effectively inhibit the release of N_2_O in the SS composting.

### 3.4 Total nitrogen

Nitrogen loss is a prominent problem in the SS composting process (Pan et al., 2018). SMS and molasses amendment significantly reduced the volatilization of NH_3_ and N_2_O in the composting, so the total nitrogen loss must be reduced in the composting. As showed in Table 3, the nitrogen loss rate of P2 treatment amended with SMS alone was 35.8%, which was 11.6% less than that in the control treatment (40.5%). While the nitrogen loss rates of P3 to P5 in the SMS and molasses treatments were 29.5%, 27.6%, and 27.4%, the nitrogen loss rate was 27.2%–32.2% lower than that of the control treatment. The main component of molasses (50%) is sucrose, so the addition of molasses could provide an easily degradable carbon source for composting microorganisms. According to the research results in author's previous literature that the easily degradable carbon source would increase the composting microbial activity and ammonia assimilation of microorganisms to reduce the volatilization of NH_3_ and N_2_O (Meng et al., 2018a). So the nitrogen loss during the composting process was reduced in the SMS and molasses treatments. Similar observations were found by Li et al. (2013) and Cai et al. (2016), and who reported that degradable carbon source amendment could decrease NH_3_ volatilization and nitrogen loss.

**Table 3:** Change of total nitrogen content and loss rate of nitrogen in five treatments.

Treatments	P1	P2	P3	P4	P5
Initial total N (%)	2.28 ± 0.02	2.71 ± 0.04	2.72 ± 0.05	2.69 ± 0.10	2.71 ± 0.05
Final total N (%)	1.52 ± 0.04	1.96 ± 0.05	2.20 ± 0.08	2.22 ± 0.07	2.23 ± 0.05
Loss rate of compost dry matter	10.7%	11.2%	12.8%	12.3%	11.8%
Nitrogen loss (%)	40.5%	35.8%	29.5%	27.6%	27.4%

### 3.5 Humification and germination index

During the SS composting process, the degradation of OM and humification process occur simultaneously. Humification process produces a typical secondary metabolite, that is humic acid, which is directly related to the stability of the composting and whose content changes reflects maturity during composting (Zhou et al., 2014). Humic acid mainly includes humic acids (HA) and fulvic acids (FA), which are composed of carbon, hydrogen, oxygen, and sulfur (Wei et al., 2007). The element composition of HA and FA is somewhat similar, besides, the contents of carbon and nitrogen of HA are higher than that of FA, while, the contents of oxygen and sulfur are lower than that of FA, which indicates that HA has higher degree of polymerization and lower degree of oxidation than FA (Zinati et al., 2001).

**Figure 4:**
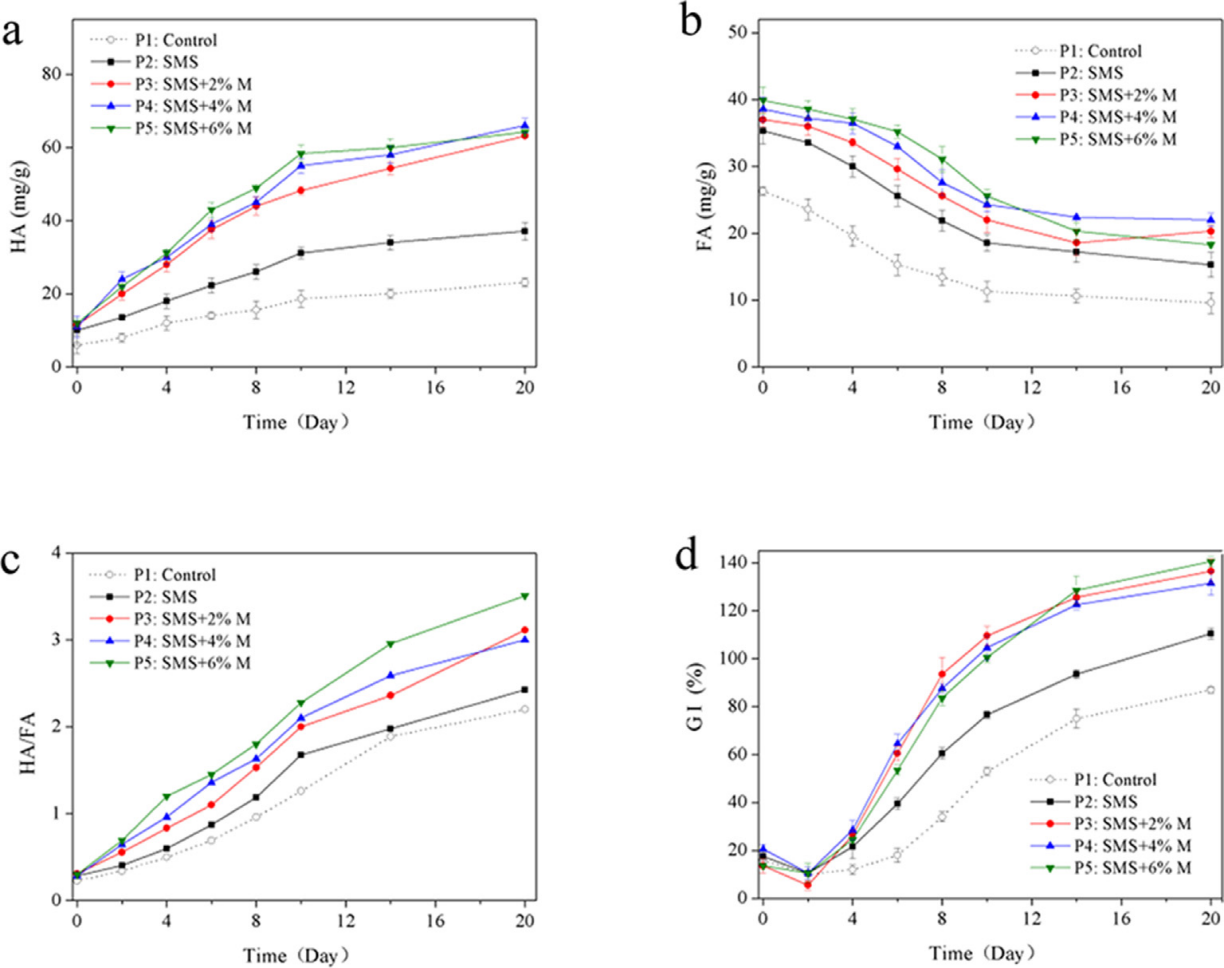
Evolution of HA (a), FA (b), HA/FA ratio (c) and GI (d) in five treatments.

Fig. 4a and b show the HA and FA contents changes of each treatment in the composting. The HA contents of all the treatments gradually risen throughout the composting process, while all the FA contents has been declining in the whole composting process. The highest HA content was 66.1 mg/g appeared in P4 treatment, followed by P5 at 64.2 mg/g, P3 at 63.2 mg/g, P2 at 37.1 mg/g, P1 at 23.1 mg/g at the end of composting, respectively. While the highest FA content was 21.9 mg/g appeared in the P4 treatment, followed by P3 at 20.3 mg/g, P518.3 at 63.2 mg/g, P2 at 15.3 mg/g, and P1 at 9.6 mg/g, respectively. Obviously, the contents of HA and FA of P1 were lower than those of SMS and molasses treatments, indicating that the addition of SMS and molasses promoted OM degradation and accelerated the humification process of composting. FA has a relatively simple structure that can be used as an energy source by composting microorganisms, so the accumulation of FA also accelerated the production rate of HA in the composting.

As shown in Figure 4c, the HA/FA ratio of all treatment showed upward trends in the whole composting process, and reached maxima at the end of composting. The highest HA/FA ratio appeared in P5 treatment was 3.51, followed by 3.09 in P3, 3.02 in P4, 2.38 in P, and 2.22 in P1, indicated that the addition of SMS and molasses increased the production of HA and promoted the polymerization of FA, concretely, improved the HA/FA ratio and the humification process of composting. One possible reason was that the addition of SMS and molasses increased microbial activity (data not shown), meanwhile, accelerated the mineralization process of OM, so increased the production rate of HA and the polymerization rate of FA.

The germination index (GI) often used to detect phytotoxicity of compost, and also often used as an important indicator for assessing composting maturity (Kosobucki et al., 2000). The evolutions of GI in the five treatments are shown in Figure 4d. The GI of all treatments showed a slight decline during the first two days of composting, this may be due to toxic substances, such as low molecular weight volatile fatty acids produced at the beginning of composting. However, with the progress of composting and the decomposition of toxic substances, the GI of each treatment increased rapidly and remained stable at the end of composting. Similar phenomena were found by (Zhang and Sun, 2017; Zhen et al., 2018), who reported that the GI remained at a low level at the beginning of composting and then gradually increased with the rise of temperature and decomposition of toxic substances until the end of composting. In addition, it is reported that the decrease of NH_4_^+^ in composting would bring about the increase of GI (Meng et al., 2018b), so the volatilization of NH_3_ would promote increase of GI.

In present study, the addition of SMS and molasses could promote the ammonia assimilation and reduce the NH_4_^+^ concentration in composting, therefore, the GI in P2~P5 amended with SMS and molasses was greater than that in P1. At the end of composting, the GI of P5 treatment reached a maximum of 141.8%, followed by P3 of 138.2% and P4 of 132.7%, and no significant difference was observed among the treatment amended with SMS and molasses. While lower GI appeared in P2 treatment amended with SMS alone was 112.3%, and the lowest GI appeared in the control treatment was 87.6%. According to the literature reported, the final GI of composting more than 80% indicate that compost product has no phytotoxicity (Unuofin and Mnkeni, 2014). In present study, P3~P5 amended with SMS and molasses reached a non-toxic state on Day 8, however, P2 and P1 reached the non-toxic state on Day 14 and Day 20, respectively. Obviously, the addition of SMS, especially the combined addition of SMS and molasses, promoted the process of composting and accelerated the maturity of composting materials.

## 4 Conclusions

The present study showed that SMS amendment, especially combination with molasses could raise composting temperature, prolong the thermophilic phase, increase CO_2_ volatilization, promote the OM degradation and humification in the SS composting. While, the difference of pH between treatments amended with SMS and molasses was not significant. Above all, the addition of SMS and molasses obviously reduced the volatilization of NH_3_, N_2_O and nitrogen loss in the SS composting. In addition, it is recommended that the SMS and molasses amendment composting should be conducted at full scale in future researches.

## Declarations

### Author contribution statement

Liqiang Meng: Conceived and designed the experiments; Wrote the paper.

Weiguang Li: Conceived and designed the experiments.

Xiancheng Zhang, Yi Zhao: Performed the experiments Li Chen: Contributed reagents, materials, analysis tools or data.

Shumei Zhang: Analyzed and interpreted the data.

### Funding statement

This work was supported by the 10.13039/501100001809National Natural Science Foundation of China (51708187, 51878216), the Outstanding Youth Foundation of Heilongjiang Sciences Academy (CXJQ2018SW01) and the Pre-research Project of Heilongjiang Sciences Academy (YY2020SW01).

### Competing interest statement

The authors declare no conflict of interest.

### Additional information

No additional information is available for this paper.
